# Serine proteases profiles of *Leishmania *(*Viannia*)* braziliensis* clinical isolates with distinct susceptibilities to antimony

**DOI:** 10.1038/s41598-021-93665-z

**Published:** 2021-07-09

**Authors:** Anabel Zabala-Peñafiel, Geovane Dias-Lopes, Léa Cysne-Finkelstein, Fátima Conceição-Silva, Luciana de Freitas Campos Miranda, Aline Fagundes, Armando de Oliveira Schubach, Maria Inês Fernandes Pimentel, Franklin Souza-Silva, Lucas de Almeida Machado, Carlos Roberto Alves

**Affiliations:** 1 Laboratório de Biologia Molecular e Doenças Endêmicas, Instituto Oswaldo Cruz, Fundação Oswaldo Cruz, Avenida Brasil, 4365, Rio de Janeiro, RJ CEP 21040-900 Brasil; 2 Laboratório de Imunoparasitologia, Instituto Oswaldo Cruz, Fundação Oswaldo Cruz, Rio de Janeiro, Brasil; 3 Laboratório de Pesquisa Clínica e Vigilância em Leishmanioses, Instituto Nacional de Infectologia Evandro Chagas, Fundação Oswaldo Cruz, Rio de Janeiro, Brasil; 4 Laboratório de Genômica Funcional e Bioinformática, Instituto Oswaldo Cruz, Fundação Oswaldo Cruz, Rio de Janeiro, Brasil

**Keywords:** Parasitology, Parasite host response, Enzymes, Proteases

## Abstract

Glucantime (Sb^V^) is the first-line treatment against American Tegumentary Leishmaniasis. Resistance cases to this drug have been reported and related to host characteristics and parasite phenotypes. In this study, 12 *Leishmania *(*Viannia*)* braziliensis* isolates from patients that presented clinical cure (Responders—R) and relapse or therapeutic failure (Non-responders—NR) after treatment with antimony, were analyzed. These parasites were assessed by in vitro susceptibility to Sb^III^ and Sb^V^, serine proteases activity measured with substrate (z-FR-AMC) and specific inhibitors (TLCK, AEBSF and PMSF). In vitro susceptibility of axenic amastigotes to Sb^III^ showed a significant difference between R and NR groups. The protease assays showed that TLCK inhibited almost 100% of activity in both axenic amastigotes and promastigotes while AEBSF inhibited around 70%, and PMSF showed lower inhibition of some isolates. Principal component and clustering analysis performed with these data yielded one homogeneous cluster with only NR isolates and three heterogeneous clusters with R and NR isolates. Additionally, differential expression of subtilisins (LbrM.13.0860 and LbrM.28.2570) and TXNPx (LbrM.15.1080) was evaluated in promastigotes and axenic amastigotes from both groups. The results showed a higher expression of LbrM.13.0860 and LbrM.15.1080 genes in axenic amastigotes, while LbrM.28.2570 gene had the lowest expression in all isolates, regardless of the parasite form. The data presented here show a phenotypic heterogeneity among the parasites, suggesting that exploration of in vitro phenotypes based on Sb^III^ and serine proteases profiles can aid in the characterization of *L. *(*V.*)* braziliensis* clinical isolates.

## Introduction

Leishmaniasis is a neglected tropical disease that affects around 15 million people worldwide, causing 40,000 deaths per year^1^. *Leishmania* spp. are eukaryotic protozoan parasites responsible for a broad range of clinical manifestations, such as cutaneous, mucocutaneous and visceral forms, which depend on the parasite species and immune state of the mammalian host^2^. In the American continent, *Leishmania mexicana* complex and subgenus *Viannia* species cause cutaneous and mucocutaneous forms that can develop into localized, disseminated or diffuse lesions, which is known as American Tegumentary leishmaniasis (ATL)^3^.

A pentavalent antimony formulation—Glucantime (Sb^V^)—is the first-line treatment against ATL^4^. In South America, this treatment doses (low and high dose) vary according to therapeutic response patterns seen in specific geographical areas^5–7^. Its mechanism of action is still not completely understood, but, it has been well accepted the hypothesis that Sb^V^ needs to be reduced to its trivalent form (Sb^III^), either directly by the parasite or within the host macrophages, to excel leishmanicidal activity^8–10^.

Moreover, the most abundant *Leishmania* spp. thiol, trypanothione [T(SH)_2_], is part of the parasite trypanothione system which is a unique detoxification defense mechanism that relies on four key enzymes: trypanothione synthetase (TryS), trypanothione reductase (TR) and tryparedoxin (TXN) and tryparedoxin peroxidase (TXNPx)^11^. This system is involved in the detoxification of metal ions, including reduction of Sb^V^ to Sb^III^, within the macrophage phagolysosome^8,10^. It is also hypothesized that Sb^V^ reduction might happen through the oxidation of T(SH)_2_ forming a stable complex—Sb^III^ (TS)_2_—that could be pumped out of the cells through parasite membrane transporters^6–9^. Interestingly, parasite’s resistance towards antimony has been associated with increased trypanothione levels and a decreased capacity to reduce Sb^V^^12–14^. Indeed, increased abundance of TXNPx has been correlated with an enhanced thiol redox potential in antimony resistant parasites, not only in lab-generated strains^15^ but also in clinical isolates^16^.

Furthermore, some studies have shown that parasite enzymes are involved in the reduction of Sb^V^ to Sb^III^ and *Leishmania* spp. resistance towards Sb^V^^14,17,18^. Particularly, serine proteases have been extensively studied in American *Leishmania* spp. due to their functional interrelationship in parasite physiology and their potential as therapeutic targets^19–25^. Among them, subtilisins are described as modulators of the trypanothione reductase system through direct action over TXNPx^26^. Subtilisin knockout promastigotes of these parasites failed to differentiate into viable amastigotes and their TXNPx peptide abundance was decreased, which suggested that subtilisins can act as maturases of specific proteins or pathways^26^.

This study aims to contribute in the understanding of Glucantime susceptibility based on *L. *(*V.*)* braziliensis* clinical isolates. Data gathered here incorporate new evidence regarding the heterogenic profile of clinical isolates based on in vitro susceptibility towards antimony and on the serine proteases profile of these parasites. Additionally, differential expression of subtilisins (LbrM.13.0860 and LbrM.28.2570) and TXNPx (LbrM.15.1080) genes was analyzed.

## Results

### In vitro susceptibility to antimony

**Figure 1:**
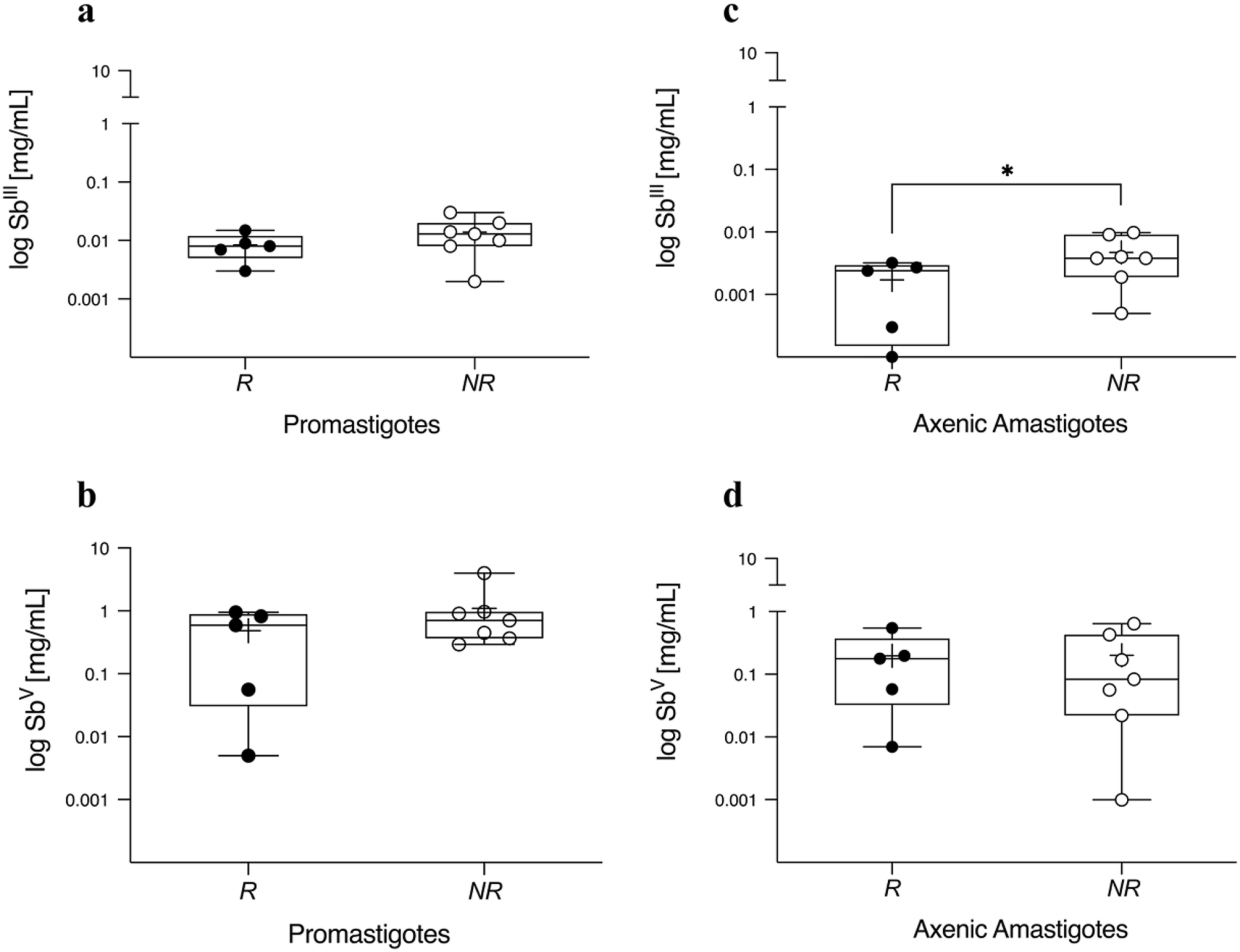
Promastigotes and axenic amastigotes in vitro susceptibility profile to antimony. Both parasite forms of each isolate (n = 12), 4 × 10^6^ promastigotes/well and 5 × 10^5^ axenic amastigotes/well were exposed to serial dilutions of trivalent (Sb^III^) and pentavalent (Sb^V^) antimonial for 48 h and 24 h in 96-well plates, respectively. The half-maximal inhibitory concentration (IC_50_ [mg/mL]) of parasites viability was measured using a fluorescence method with AlamarBlue reagent. Parasites isolated before treatment of patients with ATL cured after antimonial therapy (R: Responder, filled circle) or with poor clinical response to therapy, either therapeutic failure or relapse (NR: Non-responder, open circle). The data is presented by boxplot diagrams as the mean of three biological replicates for each isolate. Asterisks indicate statistically significant differences: *p = 0.04. The figure was generated using GraphPad Prism version 9.0.1.

Both, Sb^III^ and Sb^V^, were unable to discriminate susceptibility profiles between promastigote forms of responders (R) and non-responders (NR) isolates. The mean IC_50_ value of R versus NR group was very close which did not allow discrimination among them (Fig. 1A,B). Meanwhile, the results showed a significant difference between the axenic amastigotes grouped as R and NR exposed to Sb^III^ (*p* < 0.05, Fig. 1C). Conversely, these forms did not respond well to Sb^V^ exposure and we were not able to discriminate among the groups (Fig. 1D). The IC_50_ values and concentration–response curves are summarized in Supplementary Files 1 and 2, respectively.

### Quantification of protease activity

**Figure 2:**
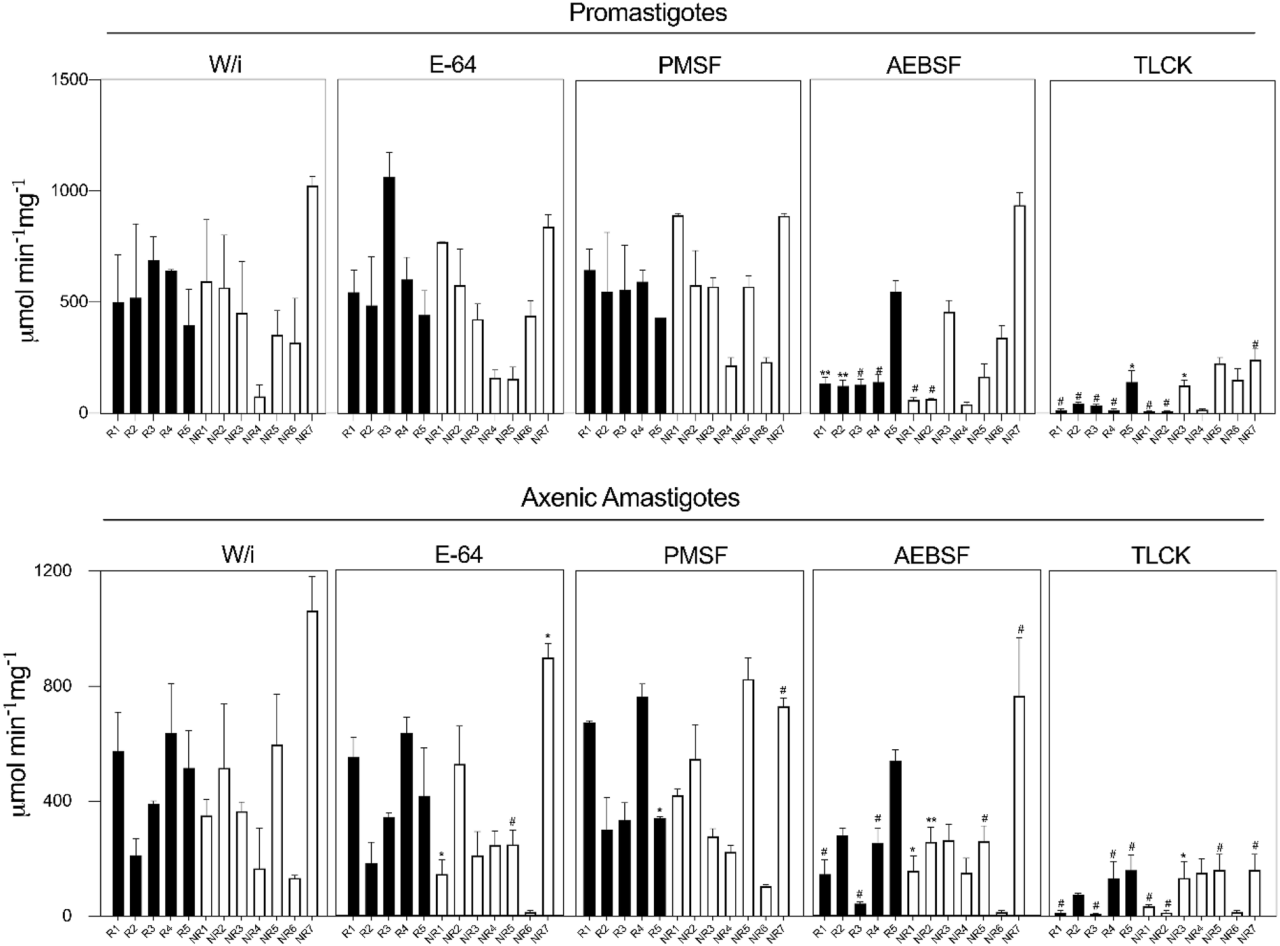
Enzymatic residual activity of whole soluble proteins from the clinical isolates. In these assays, protein extract [5 µg] of promastigotes and axenic amastigotes were measured using a specific fluorogenic peptide substrate of serine proteinases, z-FR-AMC (1 mM). The enzymatic activities (mmol min^−1^ mg of protein^−1^) were assessed without inhibitor (w/i) and in the presence of inhibitors: E-64 [10 µM], PMSF [1 mM], AEBSF [1 mM] and TLCK [100 µM]. R: Responder (black bars); NR: Non-responder (white bars). The results are shown as mean and the standard deviation (±) of three independent experiments. Asterisks indicate statistically significant differences of enzymatic activities in the absence (w/i) and presence (E-64, PMSF, AEBSF, TLCK) of inhibitors for each isolate: *p = 0.01; **p = 0.005; ^#^*p* ≤ 0.0005. The figure was generated using GraphPad Prism version 9.0.1.

All analyzed samples presented protease activity, as they were able to hydrolyze the Z-FR-AMC substrate, a chromogenic synthetic peptide appropriate for serine proteases, such as cathepsins, kallikrein and plasmin^27^. Promastigotes had a fairly similar activity without inhibition (W/i) between promastigotes (76 ± 0.07 to 1025 ± 0.07 µmol . min^-1^ mg of protein^-1^) and axenic amastigotes (133 ± 0.003 to 1063 ± 1.2 µmol . min^-1^ mg of protein^-1^), (Fig. 2 and Supplementary File 3). The specificity of serine proteases activity was assessed by measuring the hydrolysis of the Z-FR-AMC substrate in the presence of selective inhibitors (Fig. 2 and Supplementary File 3). In general, enzymatic activities in presence of TLCK were significantly lower compared to the other serine proteases inhibitors. For promastigotes, the residual activities ranged from 9 ± 0.03 to 241 ± 0.1 µmol min^−1^ mg of protein^−1^, while for axenic amastigotes from 8 ± 0.04 to 161 ± 0.09 µmol min^−1^ mg of protein^−1^. Additionally, AEBSF in promastigotes ranged from 40 ± 0.03 to 937 ± 0.09 µmol min^−1^ mg of protein^−1^, and in axenic amastigotes ranged from 14 ± 0.04 to 766 ± 0.1 µmol min^−1^ mg of protein^−1^. Conversely, PMSF residual activities were the highest among all isolates, (Fig. 2).

Furthermore, inhibition assays using E-64, a cysteine protease inhibitor, suggests that, under our controlled conditions, Z-FR-AMC was not cleaved by cysteine proteases from the isolates’ protein extracts, (Fig. 2). The majority of residual activities were similar or even higher than those W/i. Exceptionally, only the axenic amastigote extracts from NR1, NR5 and NR7 showed a significant difference when compared to their respective W/i controls.

The inhibition percentage of each serine inhibitor was calculated using each isolate W/i as a comparison (Supplementary File 4). TLCK showed the strongest inhibition with almost 100% of inhibition for R1, R2, R3, R4, NR1 and NR2 both in promastigotes and axenic amastigotes extracts. Similarly, AEBSF strongly inhibited the same group of isolates (70%) but only for the promastigotes’ extracts, while for the axenic amastigotes extracts the inhibition was dispersed, only R1, R3 and NR6 were 70% inhibited. On the contrary, PMSF showed the lowest inhibition rates. In promastigotes extracts of R3, R4, NR6 and NR7, and axenic amastigotes extracts of R3, R5, NR3, NR6 and NR7 it inhibited less than 35%. Altogether these results indicated that these isolates have a distinct quantitative profile for serine protease activities in both assayed parasite forms.

### Cluster analysis

**Figure 3:**
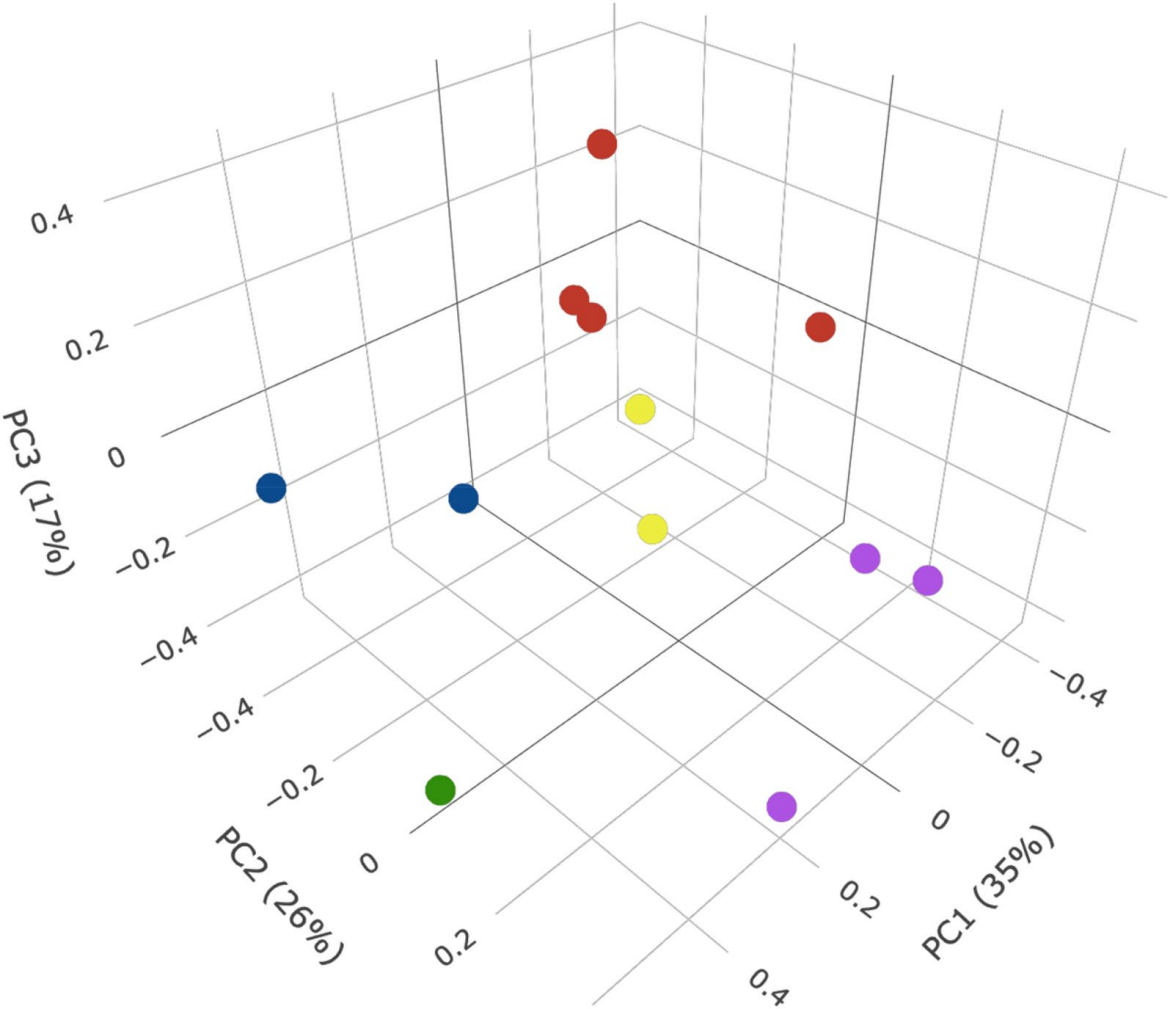
Cluster analysis of *L. *(*V.*)* braziliensis* clinical isolates. Principal components analysis (PCA) was performed to group clinical isolates based on the normalized quantitative variables. Each point represents the first three principal components of a clinical isolate. The points are colored according to the cluster they belong to, C1 (blue filled circle): NR6 and R3; C2 (red filled circle): R1, R3, NR5 and NR7; C3 (green filled circle): NR3; C4 (violet filled circle): NR2, R2 and R4; C5 (yellow filled circle): NR1 and NR4. The figure was generated using R version 1.4.1106.

The first three PCs explained approximately 80% of the data variance. Based on our analysis of the total sum of squares as a function of the number of clusters (Supplementary File 5) we obtained five clusters: Cluster 1, Cluster 2, Cluster 3, Cluster 4, and Cluster 5 (Fig. 3). There was one cluster that contained, exceptionally, only one clinical isolate (Cluster 3: NR3). One homogenous cluster containing two isolates from the same clinical group (Cluster 2: NR1, NR4) while the remaining three were heterogeneous (Cluster 1: NR6, R5; Cluster 4: R1, R3, NR5, NR7; and Cluster 5: NR2, R2, R4).

In the heterogeneous Cluster 1, the distances between both members for all variables ranged from 0.2 to 0.7, except for TLCK inhibition over promastigotes (0.04) and axenic amastigotes (0.9), (Supplementary File 6). Moreover, within Cluster 4, another heterogeneous cluster with two R and two NR isolates, TLCK inhibition between R and NR was the highest for both promastigotes and axenic amastigotes, while, PMSF and AEBSF values ranged from 0.06 to 0.85 without an established pattern (Supplementary File 7). Also, in Cluster 5, a cluster containing two R and one NR isolate, there was a pattern among variables with all distance values lower than 0.56, exceptionally, TLCK inhibition of NR3 versus R6 axenic amastigotes being the highest distance (0.72), (Supplementary File 8). Regarding Sb^III^ distance within Cluster 4 and Cluster 5, there is no value higher than 0.43.

### Quantitative analysis of gene expression

**Figure 4:**
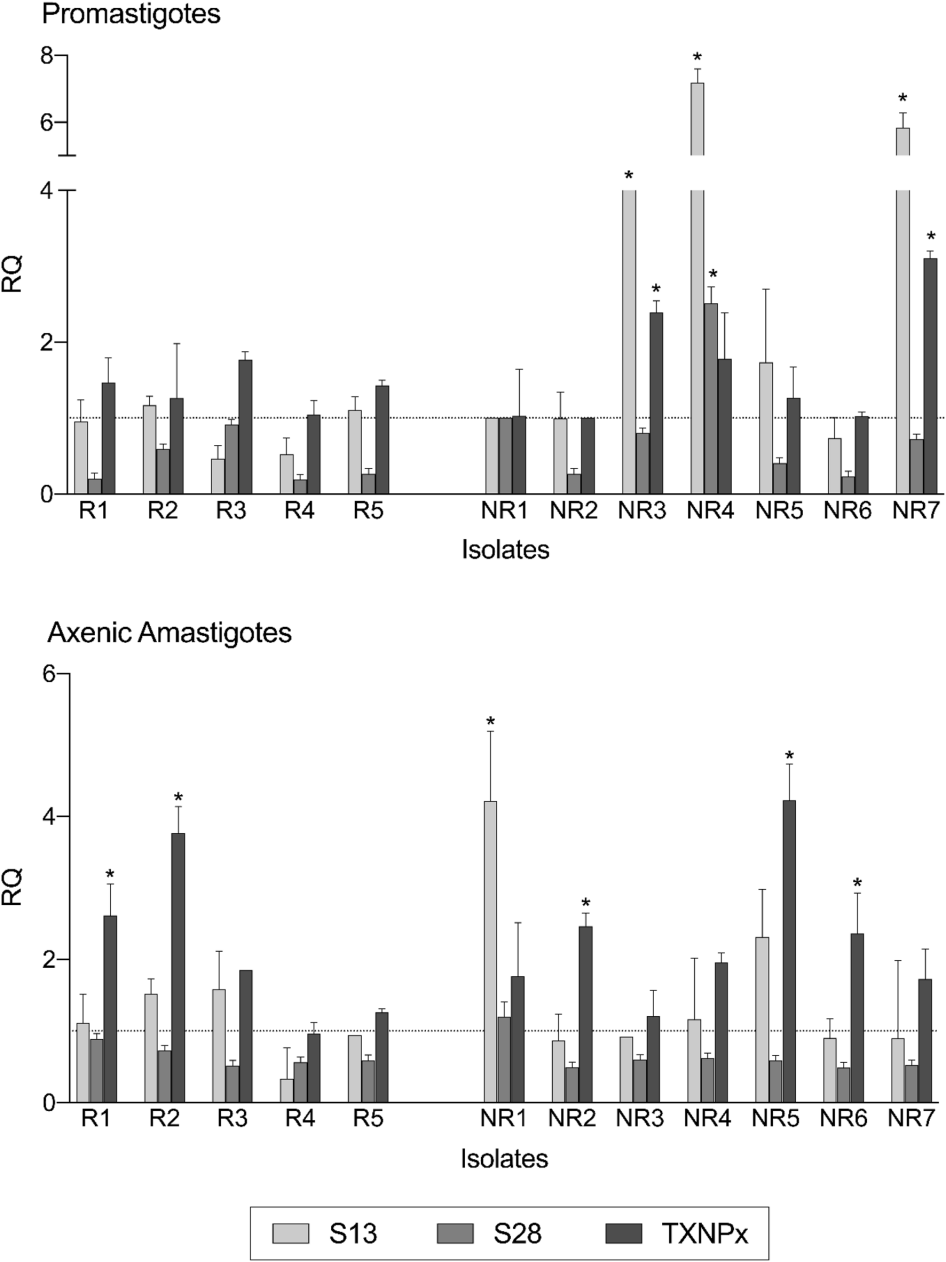
Differential gene expression of subtilisins and TXNPx of promastigotes and axenic amastigotes of *L. *(*V.*)* braziliensis* clinical isolates. Transcript levels were evaluated by qRT-PCR and the resulting relative quantification (fold-change) values of subtilisins (LbrM.13.0860, silver bars), (LbrM.28.2570, nickel bars) and TXNPx (LbrM.15.1080, iron bars) are presented. Actin and protein S8 were used as endogenous controls. The ∆∆Ct value of each gene was calculated pair-to-pair between promastigotes and axenic amastigotes, and the promastigote sample with lowest expression for each gene was set as reference sample. The dashed lines indicate RQ level^1^ of the reference samples: NR1 promastigote for subtilisins; NR2 promastigote for TXNPx. The graph presents the mean of two independent experiments performed in triplicate. *p < 0.05. The figure was generated using GraphPad Prism version 9.0.1.

Evaluation of differential gene expression between promastigotes and axenic amastigotes of *L. *(*V.*)* braziliensis* clinical isolates was based on RQ values. Data showed that all samples expressed the evaluated genes, however only promastigotes of three isolates (NR3, N4 and NR7), as well as axenic amastigote of one isolate (NR7), showed significantly higher expression of subtilisin 13 when compared to reference sample (NR1 promastigote), (Fig. 4). In the case of TXNPx expression, promastigotes of NR3 and NR7 isolates, and axenic amastigotes of R1, R2, NR2, NR5 and NR6 isolates, showed significantly higher expression when compared to reference sample (NR2 promastigote), (Fig. 4). On the other hand, the subtilisin 28 gene presented the lowest expression in all isolates, regardless of the parasite form, (Fig. 4).

## Discussion

The hypothesis that *L. (V.) braziliensis* clinical isolates have, indeed, different response profiles towards antimony was corroborated in this study by in vitro susceptibility experiments using Sb^III^. It is known that antimonial therapy failure and resistance do not only depend on host characteristics (nutrition, immune status, comorbidities, inadequate drug doses and treatment follow-up) but also on parasite factors (strains innate susceptibility, virulence factors, biologic profile)^28–32^. In this context, genome plasticity of *Leishmania* spp. with frequent copy number variations of genes or chromosomes, as well as recombination events, increases their diversity and biological capacity to expand under different stress conditions^33–35^.

Generally, the explanation of different response profiles to antimony is based on intracellular amastigotes that is the “gold standard” model to test susceptibility. However, axenic amastigotes have been used as tools to evaluate response towards Sb^V^^36–38^ as well as to Sb^III^^32^. Therefore, axenic amastigotes are a feasible model to characterize in vitro phenotypes since similarities in relation to morphology, metabolic profiles and virulence genes expression have been described in these two models^39–43^.

The in vitro Sb^III^ susceptibility of axenic amastigotes performed here was able to distinguish *L. (V.) braziliensis* clinical isolates, while this did not happen with parasites to Sb^V^. This result can be directly related to the insensitivity to Sb^V^ presented by *Leishmania* spp. promastigotes, with the exception of *Leishmania (Sauroleishmania) tarentolae*^32^. However, the Sb^V^ IC_50_ values of promastigotes obtained here are in concordance with previous findings in *L. *(*V.*)* braziliensis* promastigotes isolated from clinical samples of the same endemic area^28^. Differently from the results obtained here, another study distinguished promastigotes from poor/bad and cured/good clinical response to antimonial therapy based on Sb^V^ IC_50_ values^44^. In fact, *L. (V.) braziliensis* isolates from Rio de Janeiro municipality share common genetic traits but have different responses to Glucantime treatment^45^. Therefore, varied in vitro susceptibility showed in this study reinforces the phenotypical heterogeneity reported for this species. Since promastigotes and axenic amastigotes have different in vitro susceptibilities, led us to evaluate the possible correlation of these phenotypes with virulence factors, serine proteases, under a biochemical approach. Serine enzymatic activity is generally confirmed by using PMSF, AEBSF and TLCK inhibitors^46^. Besides, PMSF and AEBSF are known to inhibit a broad range of serine proteases including subtilisins while TLCK has a greater preference for *Leishmania* spp. oligopeptidases^21,47–49^. The inhibition profiles found here suggest that there are different groups or isoforms of serine proteases in each clinical isolate, and its respective biological forms.

In this study, cluster analysis was performed to investigate if the in vitro phenotypes correlate with the clinical response of each clinical isolate. The presence of heterogeneous clusters was better understood once we compared the pairwise distance between the members of each cluster. This analysis showed a similar pattern of PMSF and AEBSF inhibition over both parasite biological forms while TLCK inhibition was significantly more varied among the heterogeneous clusters. This observation supports the hypothesis that parasites of the subgenus *Viannia* are a polyclonal population with high genetic variability and, consequently, phenotypic diversity^40,50^. Other studies have shown that genetic and phenotypic characteristics among different *L. (V.) braziliensis* strains are associated with different clinical manifestations and drug resistance^45,51^. Therefore, *L. *(*V.*)* braziliensis* isolates can be composed of distinct enzymatic profiles that influence host-parasite interactions and, consequently, the success or failure of specific drugs, as well as amplifying a scenario of possibilities about the influence of these enzymes in the resistance mechanisms.

The elucidation of antimony resistance mechanisms, and the search for biomarkers, present severe challenges since drug resistance is a multifactorial phenomenon and the genetic variability found in *Leishmania* spp. clinical isolates. can increase this complexity^52–54^. This panorama is aggravated by therapeutic regimens with the first-line drug that varies from a high (20 mg/kg/day) to a low dose (5 mg/kg/day) in Brazil’ endemic areas, since response to treatment with antimonial drugs varies depending on the parasite strain involved^55^. This complexity is evidenced by the existence of responder and non-responder patients to the therapeutic regimen with Glucantime at low doses, as is the case of clinical isolates from Rio de Janeiro municipality^28,44^, showing a heterogenic profile within the same endemic area.

This heterogenic profile is evidenced in our Sb^III^ susceptibilities assays with axenic amastigotes, which showed that IC_50_ differences between R and NR group are small, and some values are similar for isolates from responder and non-responder patients (R1, R4 and NR7; R2, NR2, NR6). These similarities could explain the heterogeneous clusters identified in our analysis. Remarkably, the two NR isolates with the highest IC_50_ values belong to the same group in the PCA analyzes, which indicates a similar profile of the biological characteristics analyzed (Cluster 2). Thus, future studies with a larger number of isolates, and *L. *(*V.*)* braziliensis*-infected patients under different therapeutic regimens, can contribute to deepen the analysis performed here. It is necessary to emphasize that studies on serine proteases role have been carried out only in laboratory strains^21,22,26,46^, therefore, evaluating them in clinical isolates can improve the knowledge of these enzymes.

In addition to the biochemical approach, this study evaluated differential gene expression of subtilisins and TXNPx. The qRT-PCR approach used was based on previous findings regarding subtilisins as maturases of TXNPx^26^ as well as TXNPx higher expression levels in antimony resistant *L. (L.) donovani* clinical isolates^16^ and *L. *(*V.*)* braziliensis* laboratory strains^56^. Our results showed that *L. *(*V.*)* braziliensis* clinical isolates expressed subtilisins and TXNPx genes and its expression level varied depending on the isolate. However, promastigotes and axenic amastigotes of all NR isolates showed increased subtilisin (LbrM.13.0860) or TXNPx (LbrM.15.1080) expression. Since antimony resistance can be related to other processes, such as drug uptake and efflux, it is recommended to evaluate a broad set of molecular markers in resistance predictive assays using clinical isolates^54^. Thus, our results suggest that subtilisin (LbrM.13.0860) could be an additional marker during molecular characterizations of *L. *(*V.*)* braziliensis*. Moreover, the serine proteases assessment approaches of this study reinforce the evidence that these proteases might be tools to characterize phenotypes, as well as to open new perspectives for the functional research of these enzymes in *Leishmania* spp.

## Methods

### Chemical, molecular biology and culture media reagents

Antibiotics (penicillin and streptomycin), Schneider’s insect medium, bovine serum albumin (BSA), Fluorogenic peptide substrate [N-benzyloxycarbonyl-l-phenylalanyl-l-arginine 7-amino-4-methylcoumarin (Z-FR-AMC)], Proteases inhibitors [trans-epoxysuccinyl-l-leucylamido(4-guanidino)butane (E-64), Phenylmethylsulfonyl fluoride (PMSF), 4-(2-Aminoethyl)benzenesulfonyl fluoride hydrochloride (AEBSF), Tosyl-L-lysyl-chloromethane hydrochloride (TLCK)], Antimony potassium tartrate (Sb^III^) were purchased from Sigma Aldrich Chemical Co. (USA). TRIzol RNA Isolation Reagent (TRIzol), RNase Henzyme, DEPC-treated water, deoxyribonucleotide phosphate solution (dNTPs), SuperScript III First-strand cDNA Synthesis Kit (SuperScript III kit), Platinum Taq DNA Polymerase, POWER SYBR Green PCR Master Mix and AlamarBlue cell viability reagent was purchased from Life Technologies/Thermo Fisher Scientific (USA). Fetal Bovine Serum (FBS) was purchased from Gibco/Thermo Fisher Scientific (Brasil). Glucantime (used as source of Sb^V^) was kindly provided by Dr. Armando de Oliveira Schubach team (INI—Fiocruz). All reagents were of analytical or superior grade.

### Statement

The protozoa isolate samples used in this research are registered on the Brasilian SisGen number A41DBDD, in compliance with the national law number 13.123 / 2015 and its regulations. No other institutional or ethical licenses were necessary to use the mentioned samples. In addition, all experimental protocols were carried out in accordance with the relevant guidelines and regulations registered in previous publications of this manuscript authors.

### Parasite samples

Twelve *L. *(*V.*)* braziliensis* clinical isolates were acquired from the biological collection of the Evandro Chagas National Institute of Infectious Diseases at Fundação Oswaldo Cruz (INI—Fiocruz). All parasites were isolated before Glucantime treatment from five patients who were classified as responders (R) since they presented complete lesion healing and clinical cure; and seven non-responders (NR) who had therapeutic failure or relapse. The medical personal of the Leishmaniasis Clinical Research and Vigilance Laboratory (LaPClinVigiLeish) at INI—Fiocruz were responsible for this classification following the criteria reported for ATL patient’s treatment in the state of Rio de Janeiro—Brasil^6,7,57^. All the isolates included in this study were previously characterized as *L. (V.) braziliensis* by multilocus enzyme electrophoresis (MLEE), according to procedures described elsewhere^58^. For this study, each isolate was named after its patient’ response: Responder (R1, R2, R3, R4, R5) and Non-responder (NR1, NR2, NR3, NR4, NR5, NR6, NR7).

### Parasites culture and in vitro differentiation

Parasites were cultured in biphasic Novy-MacNeal-Nicolle (NNN) medium with 10% of inactivated FBS. Then, they were expanded in Schneider´s insect medium at pH 7.2 supplemented with 20% of inactivated FBS, 200 IU penicillin and 200 mg/mL streptomycin and maintained at 26 °C. To obtain each isolate growth curve, 3 × 10^5^/mL promastigotes were initially cultured and maintained in 25 cm^2^ flasks containing 5 mL of the medium described above. Daily, for eight days, a 10 µL aliquot was taken to determine the number of viable parasites using a Neubauer chamber (data not shown). Each parasite isolate did not have more than 7 passages since isolation. Differentiation from promastigotes to axenic amastigotes was performed as previously described elsewhere^41^, with a few modifications. Briefly, 5 × 10^5^ per mL of log-phase promastigotes were cultured in Schneider medium (pH 5.5) supplemented with 20% of FBS, 60 IU penicillin, 60 mg/mL streptomycin and maintained at 26 °C for 2 days. Then, to complete differentiation, each culture was subjected to temperature shock at 32 °C for 2 days. After this period, full differentiation was verified under an optical microscope (Labomed, Labo America, Inc.) and these parasites—named as one-day axenic amastigotes—were immediately used for all experimental assays (Supplementary File 9).

### In vitro susceptibility assays

Log-phase promastigotes and one-day axenic amastigotes forms, of each isolate, were tested against Sb^V^ and Sb^III^ to measure the half-maximal inhibitory concentration (IC_50_) induced by each drug. The IC_50_ was determined by AlamarBlue reduction assay as previously described^59^, with some modifications. Briefly, each parasite form was seeded in 96-well plates in triplicate at adequate conditions: log-phase promastigotes (4 × 10^6^ parasites/mL) and one-day axenic amastigotes (5 × 10^5^ parasites/mL) in 0.1 mL of Schneider medium (pH 7.2 for promastigotes or pH 5.5 for axenic amastigotes) supplemented with 20% of FBS and each drug in decreasing concentrations, leaving one column without any drug to serve as the control. Sb^V^ concentrations ranged from 20 mg/mL to 6 × 10^–4^ mg/mL; Sb^III^ concentrations ranged from 0.196 mg/mL to 5 × 10^–6^ mg/mL, with a 2:1 dilution factor between each one. After parasites incubation (Promastigotes: 26 °C, 48 h; Axenic amastigotes: 32 °C, 24 h), AlamarBlue reagent was added to each well (10 µL) followed by a new incubation at their respective temperatures for 4 h. Then, each plate was read on a Spectramax 190 microplate spectrofluorometer (Molecular Devices Corporation) at 570 excitation and 590 nm emission wavelengths, and the percentage of reduction of AlamarBlue was determined.

### Protein extraction

Log-phase promastigotes (10^8^ to 10^9^ parasites/mL) and one-day axenic amastigotes (10^8^ parasites/mL) of each isolate were separately used to obtain whole protein extracts as it follows. Parasites were washed by centrifugation (3000×*g*, 4 °C, 10 min) in sterile cold PBS pH 7.2. Then, the pellets were re-suspended in 1 mL of lysis buffer (100 mM Tris–HCl pH 8.0, 150 mM NaCl, 10% glycerol and 0.6% Triton X-100) and subjected to a minimum of 5 freeze–thaw cycles. After parasites lysis, confirmed by optical microscopy, the soluble fraction was obtained by centrifugation (25 000×*g*, 4 °C, 30 min) and the supernatant stored at − 80 °C until further use. The parasites total protein concentrations were determined by the Lowry method using BSA as a standard protein^60^.

### Enzymatic assays

The serine protease activity of the whole protein extract, 5 µg of total protein, was assessed in activation buffer (Tris–HCl [10 mM], pH 7.5) using a specific fluorogenic peptide substrate, Z-FR-AMC [1 mM], at a final volume of 60 μL. Samples were incubated (37 °C, 60 min), and the variance in the relative fluorescence was monitored on a Molecular Devices SpectraMax spectrophotometer (Gemini XPS).

Inhibition assays were performed by incubation (25 °C, 5 min) of each sample with specific inhibitors of proteases: E-64 [10 mM] (for cysteine proteases), PMSF [1 mM] (for serine- and cysteine proteases), AEBSF [1 mM] (for serine proteases such as trypsin, chymotrypsin, plasmin, kallikrein and thrombin), and TLCK [100 mM] (for serine proteases such as trypsin and trypsin-like). All inhibitors were assessed at the maximum recommended concentrations^61^.

The substrate cleavage rate was defined as follows: v = Δs/Δt, where v = velocity, Δs = substrate concentration variation and Δt = total reaction time, as determined elsewhere^39^. The self-degradation of the fluorescent peptide substrate was controlled throughout the assay to avoid incorrect readings; the enzymatic activity is expressed as mmol min^−1^.mg of protein^−1^.

### Primers design

The primers used in this study were previously designed and used to detect serine proteases and tryparedoxin peroxidase transcripts^46^. Both housekeeping genes used in this study, 40S Ribosomal protein S8 and Actin, were also previously designed and analyzed for *L. *(*V.*)* braziliensis*^46,52,62^. Briefly, the design is based on the *L. *(*V.*)* braziliensis* subtilisins (Gene ID LbrM.13.0860; LbrM.28.2570, tryparedoxin peroxidase (Gene ID LbrM.15.1080, Actin housekeeping gene (Gene ID LbrM.04.1250) and 40S Ribosomal protein S8 housekeeping gene (Gene ID LbrM.24.2160) sequences recorded in the GeneDB database (http://www.genedb.org). All primers were synthesized by Invitrogen Brasil at a concentration of 50 nM and purified by desalting.

### RNA extraction and cDNA synthesis

Log-phase promastigotes (10^8^ to 10^9^ parasites/mL) and one-day axenic amastigotes (10^7^ parasites/mL) were separately lysed in 1 mL TRIzol containing 200 μL of chloroform. For RNA extraction the samples were centrifuged (10,000×*g*, 4 °C, 10 min) and the supernatants containing RNA were dissolved in Isopropanol (12,000×*g*, 4 °C, 20 min) and washed with 70% ethanol (9000×*g*, 4 °C, 5 min). Then, each pellet was re-suspended in 30 to 40 μL of DEPC-treated water and incubated (56 °C, 10 min) to complete dissolution. The RNA concentrations were measured by spectrophotometry at 260/280 nm and 230/260 nm. DNAse treatment and cDNA synthesis were performed using the SuperScript III Kit with a maximum of 4 µg of total RNA. cDNA concentration of each sample was measured with Qubit ssDNA Assay Kit (Thermo Fisher Scientific), following the manufacturer’s protocol.

### Real time reverse transcription polymerase chain reaction (qRT-PCR)

For real-time PCR assays, 2 μL cDNA (at 1 ng/μL) were used in a final reaction volume of 8 μL, with Power SYBR Green PCR Master Mix 1X, 3 μM of forward and 3 μM of reverse primers, in a ViiA7 Real-Time PCR System (Applied Biosystems, Foster City, CA, USA), in 384 well plates. PCR cycling conditions were: a first step at 95 °C for 10 min, followed by 40 cycles at 95 °C for 15 s and 56 °C for 1 min. To check for the primers specificity, melting curves were generated after the 40 cycles at 95 °C for 15 s, 60 °C for 1 min and 95 °C for 15 s. Gene expression was calculated by relative quantitation using the comparative Ct method (ΔΔCt), as previously described^63^, with threshold set at 0.04. As endogenous genes, the housekeeping Actin and protein S8 genes were used. Gene expression was expressed as fold change or RQ values (2^-ΔΔCt^), in relation to sample with the lowest expression for each evaluated gene (from promastigotes), used as reference sample. The primers sequences and details of standard curve parameters for gene expression are presented in Supplementary File 10.

### Statistical analysis

To calculate IC_50_ values and establish significant differences we used Student’s T-test, considering *p* < 0.05 as a significant difference. While, to compare inhibition regimens (W/i, E-64, PMSF, AEBSF, TLCK) in each isolate, and to determine significant differences between gene expression levels of each isolate versus each gene reference sample we used 2way ANOVA followed by Dunnett’s multiple comparison test. All these tests were performed using GraphPad Prism version 9.0.1 for macOS (GraphPad Software, San Diego, California USA, www.graphpad.com). Additionally, the quantitative variables obtained for each clinical isolate: (i) promastigotes Sb^V^ IC_50_, (ii) promastigotes Sb^III^ IC_50_ and (iii) axenic amastigotes, (iv) Z-FR-AMC protease activity substrate over promastigotes and (v) axenic amastigotes, (vi) PMSF inhibition over promastigotes and (vii) axenic amastigotes, (viii) AEBSF inhibition over promastigotes and (ix) axenic amastigotes, (x) TLCK inhibition over promastigotes and (xi) axenic amastigotes were normalized and subjected to principal component analysis (PCA). Here we have 11 variables per isolate and biological form which is why PCA analysis helped us reduce dimensionality and perform further cluster analysis^64^. The first three PCs (PC1, PC2, PC3) were used to cluster the clinical isolates using the K-means algorithm clustering method. To determine the optimal number of clusters we used the total-within cluster sum of squares (twcss), as a function of the number of clusters, where the squared distances between each cluster centroid (${\bar{\mathrm{x}}}_{{\mathrm{c}}_{\mathrm{i}}}$) and each of its cluster members ($\mathrm{x}, \mathrm{where} \mathrm{x}\in {\mathrm{c}}_{\mathrm{i}})$ are summed over each cluster ${\mathrm{c}}_{\mathrm{i}}$, Nc is the total number of clusters, Eq. (1). Additionally, with the previously normalized data, we explored the characteristics of the clusters by calculating the pairwise distance between clusters using the normalized values of Sb^III^ IC_50_, PMSF, AEBSF and TLCK inhibition over promastigotes and axenic amastigotes. These statistical analyses were carried out using R (version 1.4.1106)^65^.

1 $${\mathrm{W}}{\mathrm{C}}{\mathrm{S}}{\mathrm{S}}=\sum\nolimits _{\mathrm{i}=1}^{\mathrm{Nc}}\sum\nolimits_{{\mathrm{x}}\in {\mathrm{c}}_{\mathrm{i}}}{{\mathrm{d}}\left({\mathrm{x}},{{{\bar{\mathrm{x}}}}}_{{\mathrm{c}}_{\mathrm{i}}}\right)}^{2}$$

## Supplementary Information

Supplementary Information.
