# Supplementary material for: Serine proteases profiles of Leishmania (Viannia) braziliensis clinical isolates with distinct susceptibilities to antimony
Source: Sci Rep. 2021 Jul 9;11:14234. doi: 10.1038/s41598-021-93665-z (PMC8271011; doi:10.1038/s41598-021-93665-z)
Supplement: Supplementary file 1 — Supplementary Information. [file 41598_2021_93665_MOESM1_ESM.docx]

**Title**

Serine proteases profiles of *Leishmania (Viannia) braziliensis* clinical isolates with distinct susceptibilities to antimony

**Author names**

Anabel Zabala-Peñafiel^1^, Geovane Dias-Lopes^1^, Léa Cysne-Finkelstein^2^, Fátima Conceição-Silva^2^, Luciana de Freitas Campos Miranda^3^, Aline Fagundes^3^, Armando de Oliveira Schubach^3^, Maria Ines Fernandes Pimentel^3^, Franklin Souza-Silva^1^, Lucas de Almeida Machado^4^, Carlos Roberto Alves^1^*

**Author affiliations**

^1^Laboratório de Biologia Molecular e Doenças Endêmicas, Instituto Oswaldo Cruz, Fundação Oswaldo Cruz, Rio de Janeiro, Brasil.

^2^Laboratório de Imunoparasitologia, Instituto Oswaldo Cruz, Fundação Oswaldo Cruz, Rio de Janeiro, Brasil.

^3^Laboratório de Pesquisa Clínica e Vigilância em Leishmanioses, Instituto Nacional de Infectologia Evandro Chagas, Fundação Oswaldo Cruz, Rio de Janeiro, Brasil.

^4^Laboratório de Genômica Funcional e Bioinformática, Instituto Oswaldo Cruz, Fundação Oswaldo Cruz, Rio de Janeiro, Brasil

***Corresponding author**

Carlos Roberto Alves – [calves@ioc.fiocruz.br](mailto:calves@ioc.fiocruz.br)

Fundação Oswaldo Cruz, Instituto Oswaldo Cruz, Laboratório de Biologia Molecular e Doenças Endêmicas, Avenida Brasil, 4365, CEP 21040-900, Rio de Janeiro, RJ, Brasil.

Supplementary file 1. *In vitro* susceptibility of clinical isolates to trivalent and pentavalent antimony

| *Clinical isolates* | IC_50_ [mg/mL]* | | | |
| --- | --- | --- | --- | --- |
|  | *Promastigotes*** | | *Axenic amastigotes**** | |
|  | Sb^V^ | Sb^III^ | Sb^V^ | Sb^III^ |
| *R1* | 0.005 ± 0.27 | 0.008 ± 0.0008 | 0.007 ± 0.16 | 0.002 ± 0.05 |
| *R2* | 0.821 ± 0.09 | 0.003 ± 0.0002 | 0.549 ± 0.11 | 0.003 ± 0.01 |
| *R3* | 0.951 ± 0.2 | 0.015 ± 0.0007 | 0.058 ± 0.3 | 9x10^-7^ ± 0.039 |
| *R4* | 0.591 ± 0.07 | 0.007 ± 0.0004 | 0.198 ± 0.13 | 0.0021 ± 0.005 |
| *R5* | 0.056 ± 0.14 | 0.008 ± 0.0011 | 0.178 ± 0.7 | 0.0003 ± 0.0024 |
| *NR1* | 0.908 ± 0.04 | 0.014 ± 0.0007 | 0.429 ± 0.06 | 0.009 ± 0.007 |
| *NR2* | 3.996 ± 1.4 | 0.008 ± 0.0003 | 0.084 ± 0.24 | 0.003 ± 0.006 |
| *NR3* | 0.449 ± 0.05 | 0.010 ± 0.0003 | 0.001 ± 0.4 | 0.004 ± 0.0033 |
| *NR4* | 0.972 ± 0.03 | 0.013 ± 0.0007 | 0.640 ± 0.05 | 0.009 ± 0.0004 |
| *NR5* | 0.295 ± 0.07 | 0.002 ± 0.0004 | 0.022 ± 0.07 | 0.0003 ± 0.0002 |
| *NR6* | 0.708 ± 0.09 | 0.030 ± 0.0004 | 0.169 ± 0.19 | 0.003 ± 0.0009 |
| *NR7* | 0.366 ± 0.4 | 0.020 ± 0.0011 | 0.056 ± 0.12 | 0.0019 ± 0.007 |

(*) Half maximal inhibitory concentration (IC_50_). (**) Promastigotes were exposed to serial dilutions of trivalent (Sb^III^) and pentavalent (Sb^V^) antimonial for 48 hours. (***) Axenic amastigotes were exposed to serial dilutions of Sb^III^ and Sb^v^ for 24 hours.


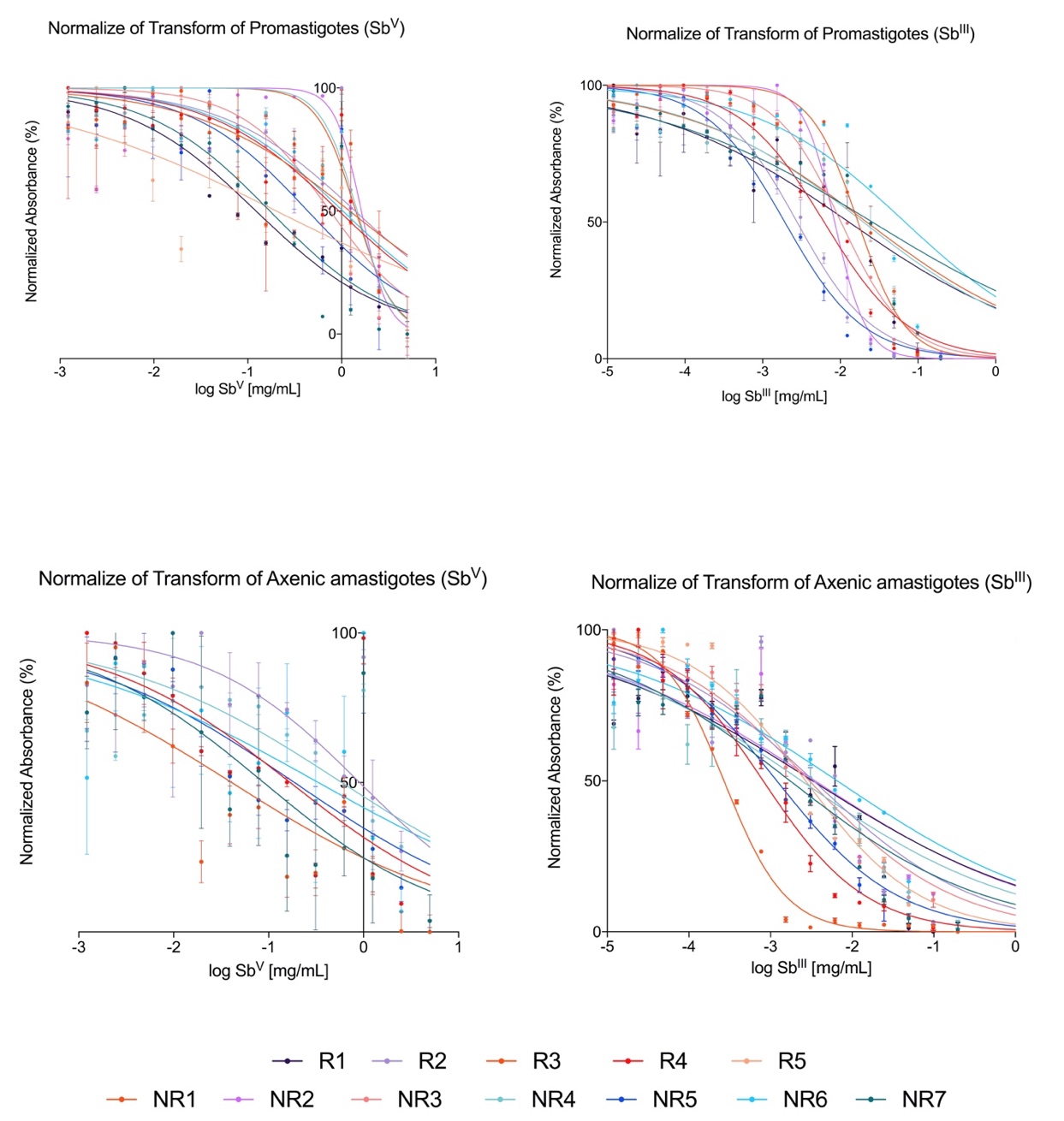


**Supplementary file 2.** Concentration-response curves to calculate IC_50._ Half maximal inhibitory concentration (IC_50_). Promastigotes were exposed to serial dilutions of trivalent antimonial (Sb^III^) for 48 hours. Axenic amastigotes were exposed to serial dilutions of Sb^III^ for 24 hours. The figure was generated using GraphPad Prism version 9.0.1.

**Supplementary file 3**. Quantification of serine proteases activity in each protein extract

| *Clinical isolates* | Enzymatic activity [μmol min-1.mg of protein-1]* | | | | | | | |
| --- | --- | --- | --- | --- | --- | --- | --- | --- |
|  | *Promastigotes^**^* | | | | *Axenic amastigotes^**^* | | | |
|  | W/i^1^ | PMSF^2^ | AEBSF^3^ | TLCK^4^ | W/i^1^ | PMSF^2^ | AEBSF^3^ | TLCK^4^ |
| *R1* | 495 ± 1.2 | 644 ± 1.2 | 133 ± 0.8 | 14 ± 0.05 | 576 ± 1.5 | 673 ± 0.02 | 146 ± 1.6 | 13 ± 0.01 |
| *R2* | 519 ± 1.7 | 548 ± 1.7 | 123 ± 0.05 | 44 ± 0.1 | 211 ± 1.2 | 301 ± 0.8 | 280 ± 0.9 | 75 ± 0.002 |
| *R3* | 690 ± 0.8 | 554 ± 1.6 | 128 ± 0.06 | 35 ± 0.09 | 391 ± 0.9 | 335 ± 0.9 | 44 ±0.09 | 8 ± 0.04 |
| *R4* | 640 ± 0.03 | 592 ± 0.7 | 140 ± 0.03 | 14 ± 0.29 | 637 ± 1.3 | 764 ± 0.2 | 256 ± 0.9 | 131 ± 0.7 |
| *R5* | 396 ± 1.5 | 429 ± 0.03 | 547 ± 0.05 | 140 ± 0.03 | 516 ± 1.5 | 346 ± 0.01 | 542 ± 0.8 | 160 ± 0.9 |
| *NR1* | 595 ± 1.5 | 890 ± 0.05 | 60 ± 0.09 | 9 ± 0.03 | 350 ± 0.9 | 421 ± 0.09 | 158 ± 1.8 | 35 ± 0.02 |
| *NR2* | 566 ± 1.2 | 576 ± 1.2 | 64 ± 0.08 | 9 ± 0.02 | 516 ± 1.9 | 547 ± 0.5 | 258 ± 1.5 | 13 ± 0.004 |
| *NR3* | 452 ± 1.7 | 570 ± 0.8 | 457 ± 0.9 | 124 ± 0.1 | 366 ± 1.1 | 278 ± 0.1 | 264 ± 1.2 | 133 ± 1.1 |
| *NR4* | 76 ± 0.07 | 215 ± 0.1 | 40 ± 0.03 | 15 ± 0.9 | 167 ± 1.8 | 224 ± 0.3 | 152 ± 0.8 | 151 ± 0.8 |
| *NR5* | 353 ± 0.9 | 571 ± 0.2 | 165 ± 0.02 | 225 ± 0.09 | 597 ± 1.6 | 825 ± 0.2 | 261 ± 1.2 | 161 ± 0.8 |
| *NR6* | 318 ± 1.2 | 230 ± 0.2 | 340 ± 0.03 | 150 ± 0.02 | 133 ± 0.03 | 104 ± 0.2 | 14 ± 0.04 | 14 ± 0.002 |
| *NR7* | 1025 ± 0.07 | 888 ± 0.09 | 937 ± 0.09 | 241 ± 0.9 | 1063 ± 1.2 | 730 ± 0.4 | 766 ± 2.1 | 161 ± 0.9 |

(*) Serine protease activity of total protein extract [5µg] assessed in activation buffer (10 mM Tris-HCl pH 7.5) using Z-FR-AMC [1 mM] as a specific fluorogenic substrate. (**) Each sample was incubated (60 min, 37 °C) and the variance in the relative fluorescence was monitored. Inhibition assays were performed by incubation (5 min, 25 °C) with (^1^) no inhibitor, (^2^) PMSF [1 mM], (^3^) AEBSF [1 mM] and (^4^) TLCK [100 μM]. R: Responder; NR: Non-responder.


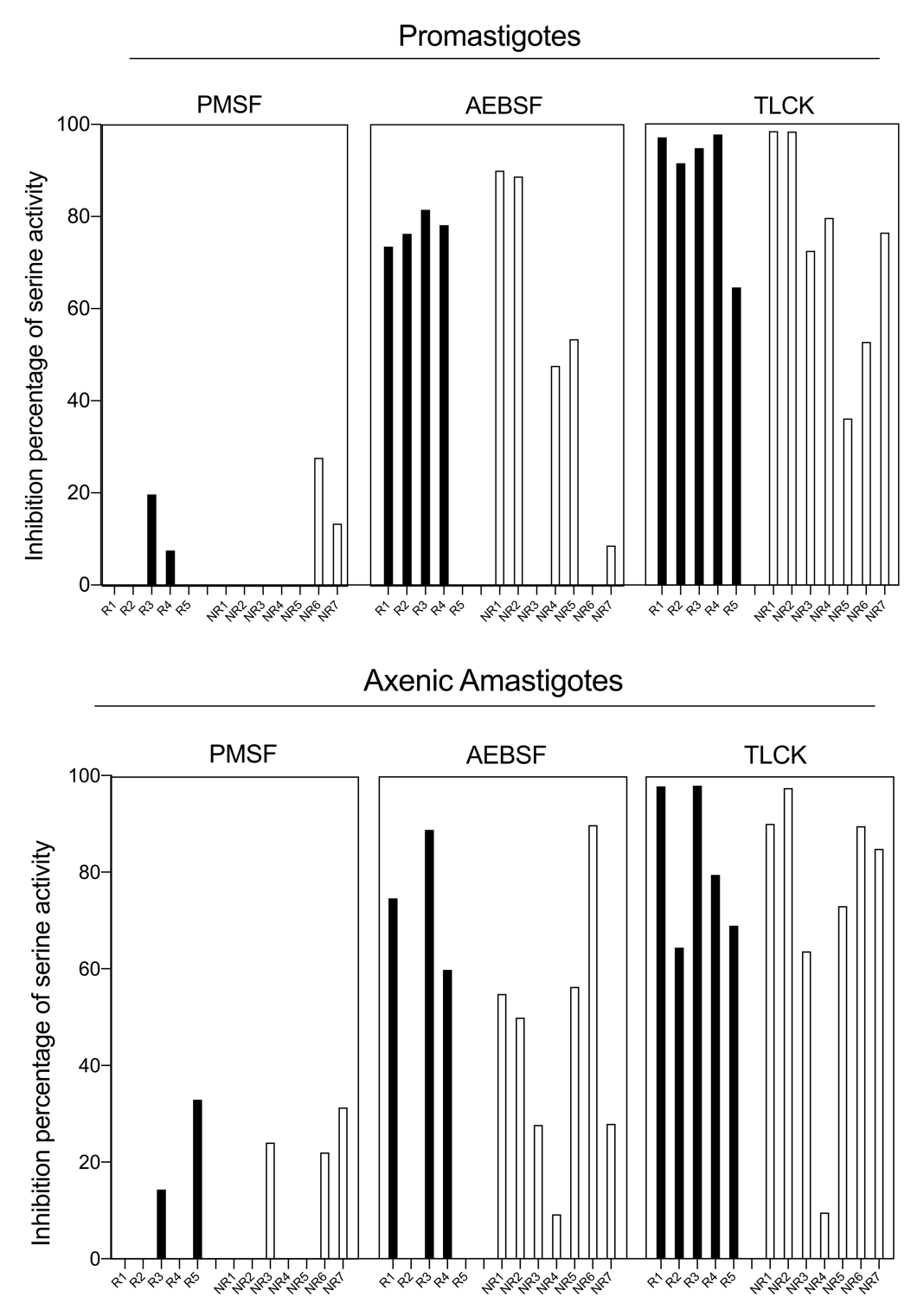


**Supplementary file 4.** Inhibition of enzymatic activity of whole soluble proteins from the isolates. The inhibition percentage of serine protases inhibitors, PMSF, AEBSF and TLCK, were calculated using each isolate value without inhibition as control – or 0% of inhibition. Parasites isolated before treatment of patients with ATL cured after antimonial therapy (R: black bars) or with poor clinical response to therapy, either therapeutic failure or relapse (NR: white bars). The figure was generated using GraphPad Prism version 9.0.1.


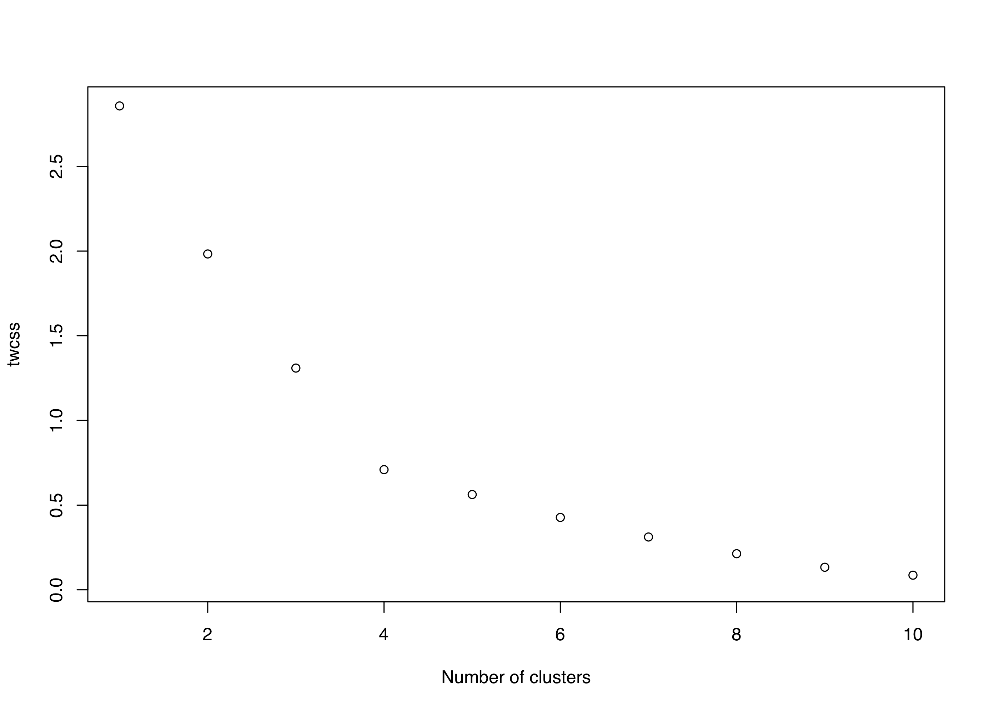


**Supplementary file 5.** The optimal number of clusters was selected after optimization of the K-means algorithm. The total-within cluster sum of squares (twcss), was analyzed to determine the number of clusters. The ideal number of clusters was determined by analysis of the elbow point (here, assumed as k = 5). The figure was generated using R version 1.4.1106.


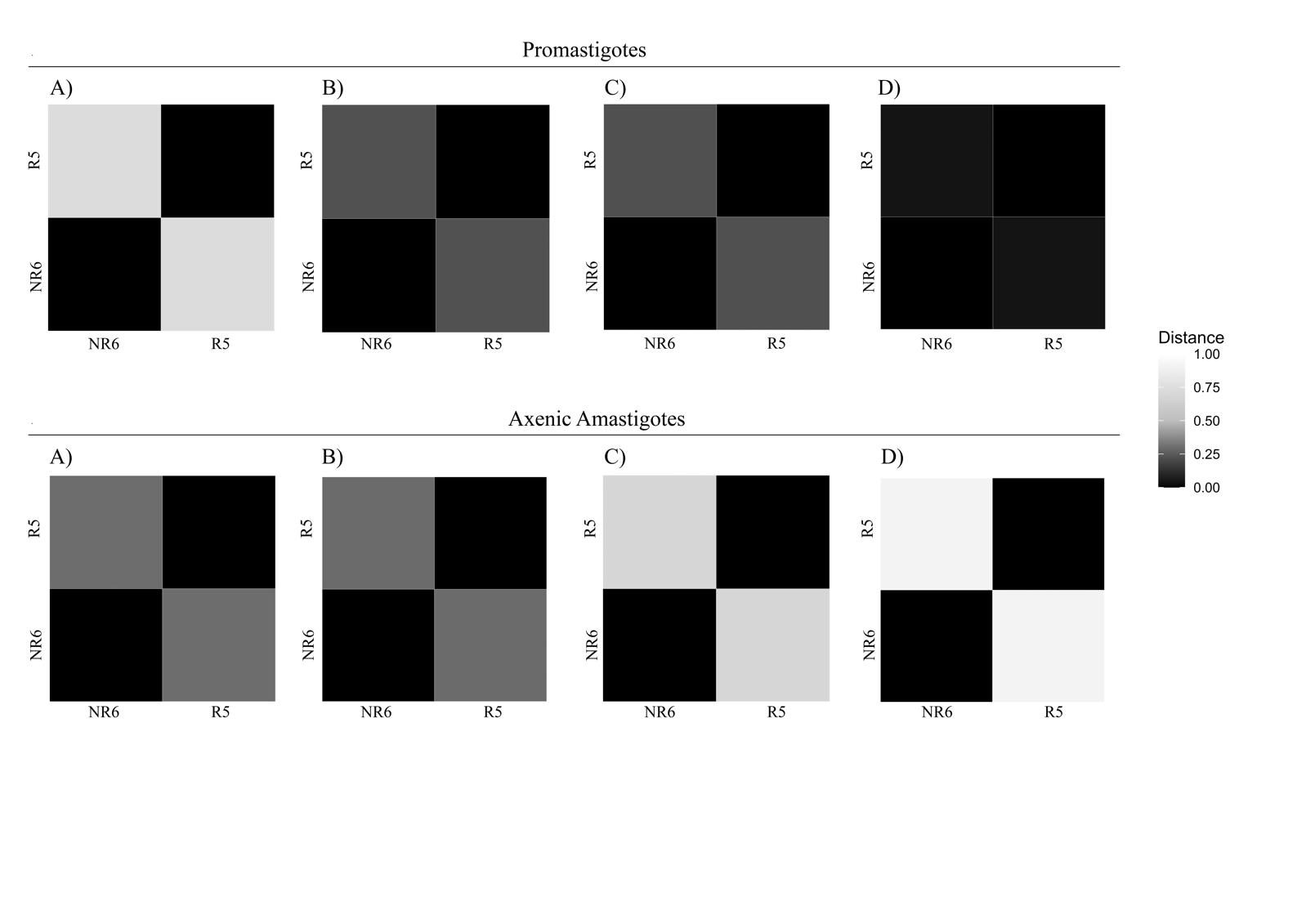


**Supplementary file 6.** Cluster 1 heatmap showing the pairwise distance of the heterogeneous clusters over four relevant biological variables. The distance represents the similarity and it ranges from 0.00 (minimum similarity) to 1.00 (maximum similarity). A) Sb^III^ [IC_50_], B) PMSF [1mM], C) AEBSF [1 mM], D) TLCK [100 µM]. R5: Responder isolate 5; NR6: Non-responder isolate 6. The figure was generated using R version 1.4.1106.


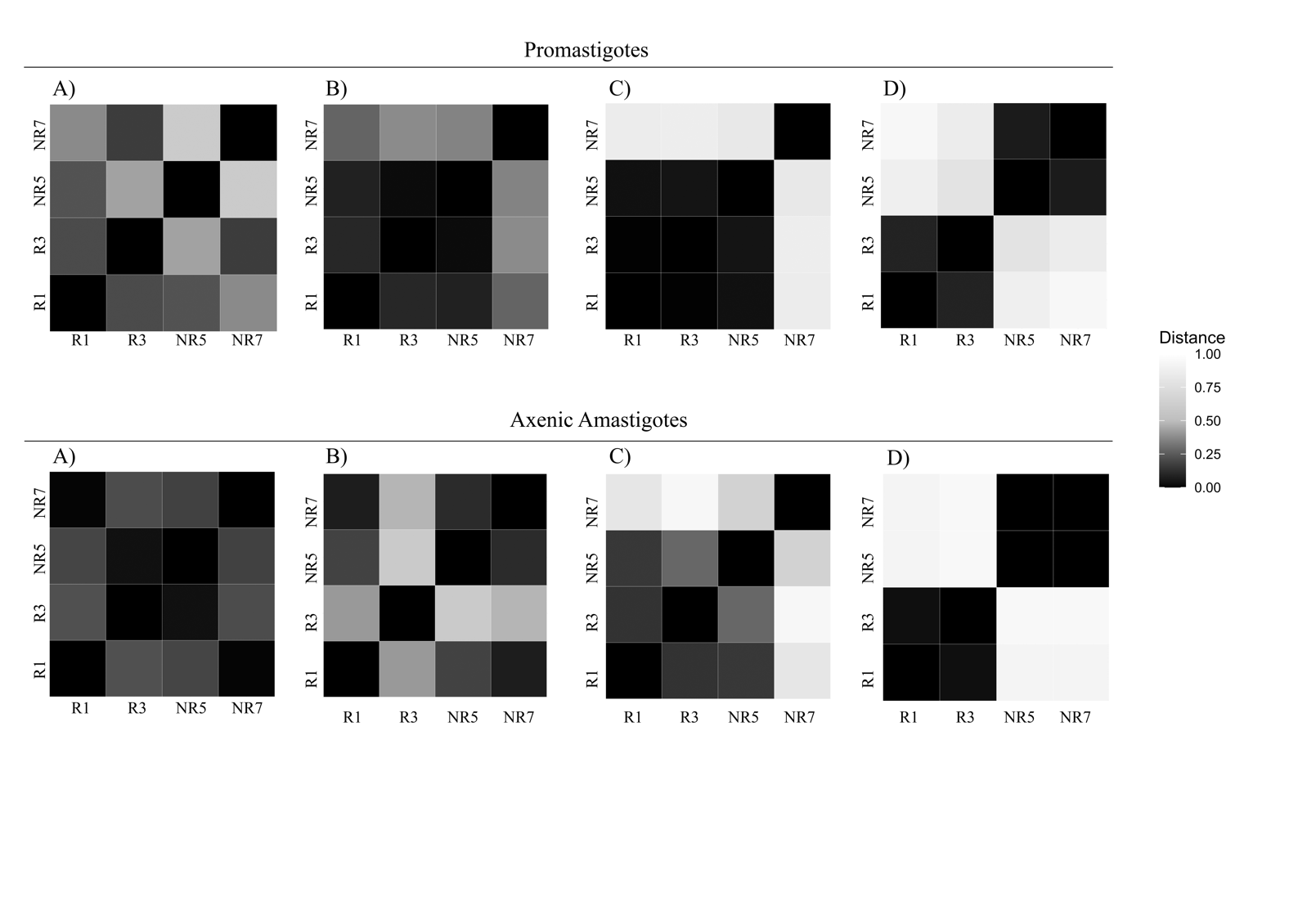


**Supplementary file 7.** Cluster 4 heatmap showing the pairwise distance of the heterogeneous clusters over four relevant biological variables. The distance represents the similarity and it ranges from 0.00 (minimum similarity) to 1.00 (maximum similarity). A) Sb^III^ [IC_50_], B) PMSF [1mM], C) AEBSF [1 mM], D) TLCK [100 µM]. R1: Responder isolate 1; R3: Responder isolate 3; NR5: Non-responder isolate 5; NR7: Non-responder isolate 7. The figure was generated using R version 1.4.1106.


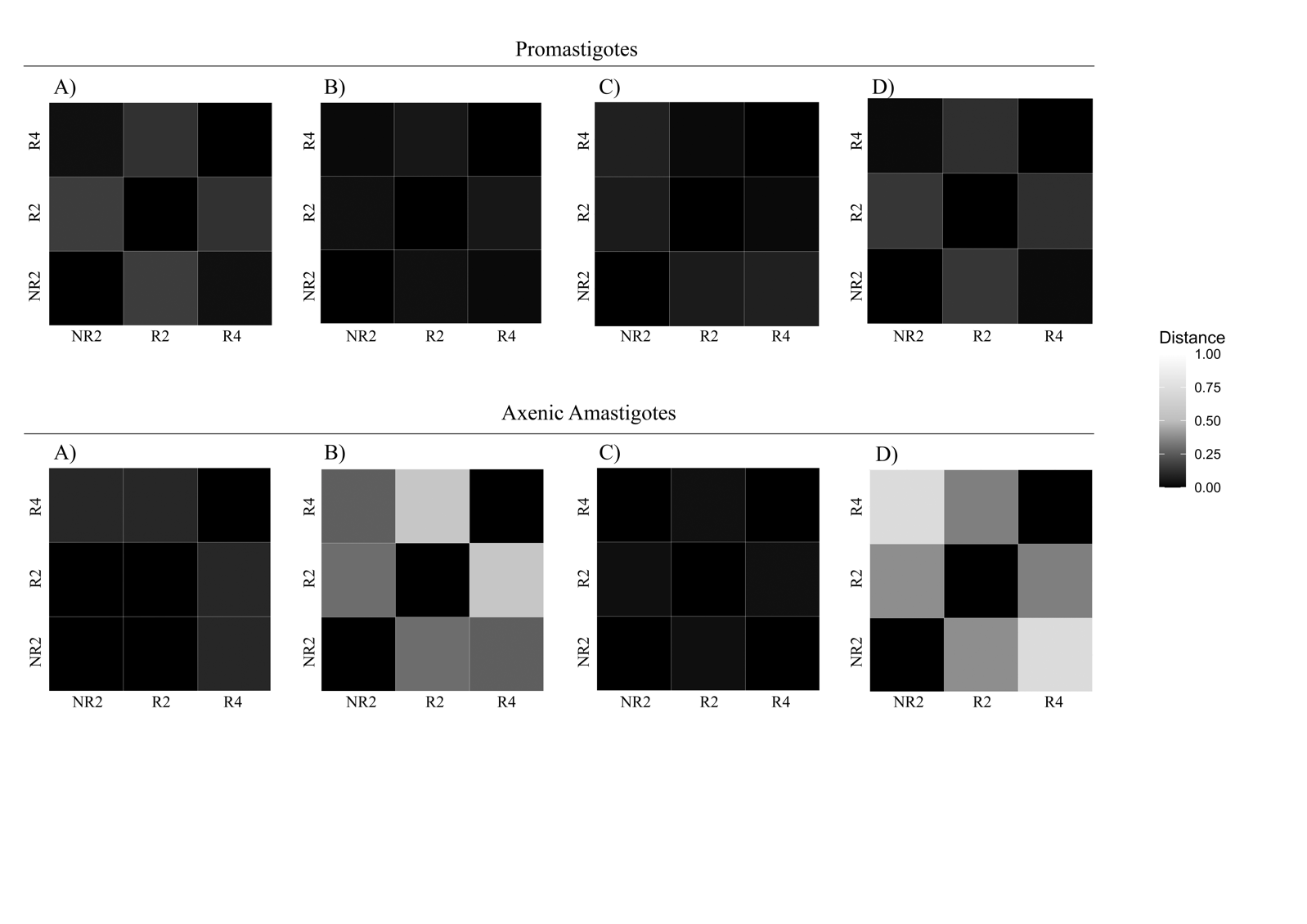


**Supplementary file 8**. Cluster 5 heatmap showing the pairwise distance of the heterogeneous clusters over four relevant biological variables. The distance represents the similarity and it ranges from 0.00 (minimum similarity) to 1.00 (maximum similarity). A) Sb^III^ [IC_50_], B) PMSF [1mM], C) AEBSF [1 mM], D) TLCK [100 µM]. R2: Responder isolate 2; R4: Responder isolate 4; NR2: Non-responder isolate 2. The figure was generated using R version 1.4.1106.


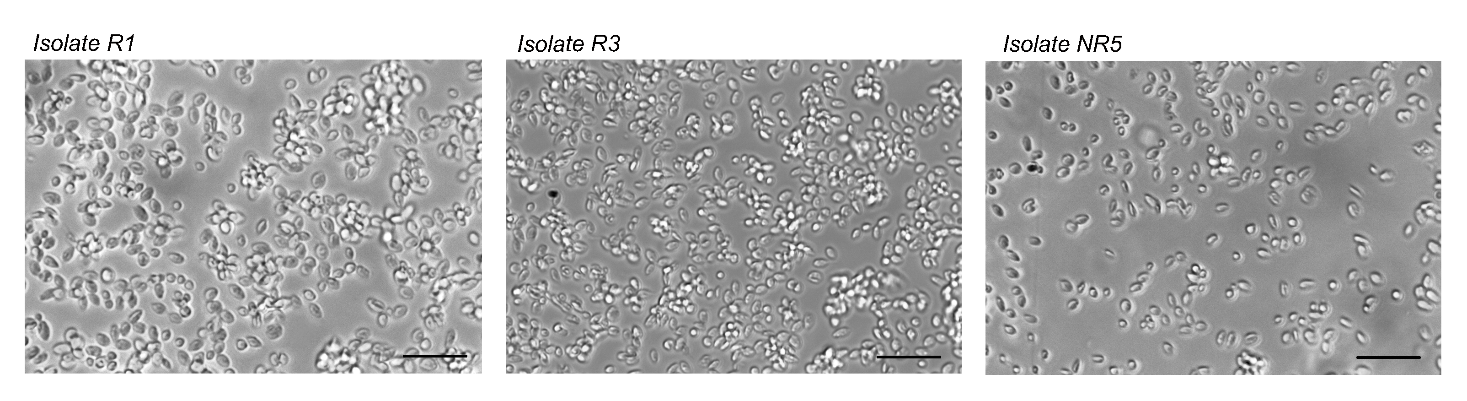
 **Supplementary file 9.** Axenic amastigotes obtained by *in vitro* differentiation. After 4 days of promastigotes to amastigotes differentiation induction, of all isolates, the parasites were observed by optical microscopy showing the expected morphological changes for *Leishmania* spp. amastigotes, such as body roundness and not apparent flagellum. The images show axenic amastigotes of three representative *L. (V.) braziliensis* clinical isolates (R1, R3 and NR5). Scale bar: 10µm.

**Supplementary file 10.** Primers sequences and standard curve parameters for gene expression of *L. (V.) braziliensis* clinical isolates

| Gene target | Primer sequences | Reference | Amplicon length | Slope | Intercept | Coefficient of linearity (r^2^) | Amplification efficiency (%) |
| --- | --- | --- | --- | --- | --- | --- | --- |
| S13 | Fw 5’-GAG CTA ACA CCA GTG GCA CA-3’  Rv 3’-ATC TGG CGA TTT CTC CCT TT-5’ | (47) | 200 bp | -3.17 | 24.00 | 0.99 | 107.04 |
| S28 | Fw 5’-CAC TGC GCT CCA CAT ACA CT-3’  Rv 3’-GCC TTC ATT CGA GCT ACA GG-5’ | (47) | 162 bp | -3.21 | 27.00 | 0.98 | 106.04 |
| TXNPx | Fw 5’-CTC TGT GGA CAG CGA GTA CG-3’  Rv 3’-TGG GGT CGA TGA TAA AGA GG-5’ | (47) | 166 bp | -3.22 | 16.93 | 0.98 | 105.46 |
| S8 | Fw 5’-GTG CGT AAC GTG AAA GAG CA-3’  Rv 3’-AAG TCG ATG CCG TAA TGC TT-5’ | (47,53,63) | 98 bp | -3.27 | 18.87 | 0.97 | 104.41 |
| Actin | Fw 5’-GTG CGT AAC GTG AAA GAG CA-3’  Rv 3’-GGC AGC TCA AAT GAC TCC TC-5’ | (47,53,63) | 100bp | -3.22 | 19.71 | 0.99 | 105.60 |
